# Zellweger Syndrome: A Case Report

**DOI:** 10.31729/jnma.8467

**Published:** 2024-02-29

**Authors:** Prajwala Yogi, Chunauti Bahik, Rahul Yadav, Puja Bhattarai, Rakshya Pandey, Sunil Raja Manandar

**Affiliations:** 1 Kathmandu Medical College and Teaching Hospital, Sinamangal, Kathmandu, Nepal; 2 Department of Pediatrics, Kathmandu Medical College ana Teaching Hospital, Sinamangal, Kathmandu, Nepal; 3 Sindhuli Hospital, Kamalamai, Sindhuli, Nepal

**Keywords:** *case reports*, *mutation*, *neonate*, *Zellweger syndrome*

## Abstract

Zellweger syndrome is an autosomal recessive disease within the spectrum of peroxisome biogenesis disorder manifesting in the neonatal period with profound dysfunction of the central nervous system, liver and kidney.Common clinical presentations include hypotonia, seizure, hepatomegaly, craniofacial dysmorphism and early death. Mutation in one of the PEX genes coding for a peroxisome assembly protein creates a functionally incompetent organelle causing accumulation of very long chain fatty acids in various organs. Here we report the case of a 5-month-old male presented at birth with hypotonia, poor feeding, gross congenital anomalies and later during early infancy with failure to thrive, several episodes of seizures, aspiration due to feeding difficulties and recurrent severe pneumonia. A whole genomic sequencing brought us to the final diagnosis of Zellweger syndrome. Despite an absence of treatment options, prompt diagnosis of Zellweger syndrome is important for providing appropriate symptomatic care, definitive genetic testing and prenatal counselling.

## INTRODUCTION

Zellweger syndrome (ZS) is a rare, fatal disorder caused by variants in PEX (peroxisome biogenesis factor 1) genes that impair peroxisome function, affecting multiple organ systems.^1^ Peroxisomes participate in various metabolic pathways such as the synthesis of ether phospholipids and bile acids, alfa and beta-oxidation of fatty acids and removal of reactive oxygen species playing a crucial role in the proper functioning of several organs.^2^ Patients in their neonatal period present with the characteristic phenotype of distinctive facial dysmorphism, pronounced hypotonia, hepatic dysfunction and seizure.^3^ The prevalence of ZS is estimated to be around 1 in 50,000 live births.^4^ This case focuses on an infant with ZS presenting with hypotonia, failure to thrive, seizure and recurrent pneumonia leading to septic shock and early death.

## CASE REPORT

A term male baby presented at birth with delayed cry after birth, neck hypotonia and gross congenital anomalies like absent ear lobule of left ear, low set ears, bilateral simian crease and undescended testis. This presentation at birth led to suspicion of Down syndrome at first. The patient was then admitted to NICU under oxygen support for a few days and a detailed workup was carried out to reach the diagnosis. Blood samples were sent for a thyroid function test which was within normal limits but the liver function test showed a moderate rise in liver transaminase enzymes. Echocardiography revealed a small patent ductus arteriosus and multiple fenestrated ASDs. USG of the abdomen and pelvis showed mild hydronephrosis of the left kidney and non-visualization of the testis in the scrotal sac and abdominal cavity. During the hospital stay the patient was kept on nil per oral for 4-5 days, later shifted to orogastric feeding and eventually days before discharge from the hospital, spoon feeding was initiated and continued as swallowing reflex was very poor. The mother gave a history of the patient becoming unresponsive due to aspiration while feeding medicine and received CPR shortly after and was admitted to a nearby hospital with a diagnosis of perinatal asphyxia and aspiration with late-onset neonatal sepsis. Several episodes of seizure were noted for which supportive management was done with antiepileptic drugs (Inj. levetiracetam and Inj. phenytoin). Due to features of severe hypotonia and muscle wasting, spinal muscle atrophy was kept in a differential. Fluorescence in situ hybridisation (FISH) was done for anomalies like trisomies 21, 13, 18 and sex chromosomes where no aneuploidy was detected. MRI of the brain revealed diffuse T2 high in intensity in subcortical and deep white matter of the bilateral cerebral hemisphere, probably an area of incomplete myelination. TORCH profiling showed CMV IgG: 4.75 (high) and HSV 1 and 2 IgG: 3.84 (high). ENT consultation was done for difficulty in swallowing and passive stimulation and breastfeeding with safe swallow strategies were advised. Then a DNA sample from the blood was sent to India for whole genomic testing which detected homozygous missense variants in the PEX gene and a final diagnosis of Zellweger syndrome was made.

**Figure 1:**
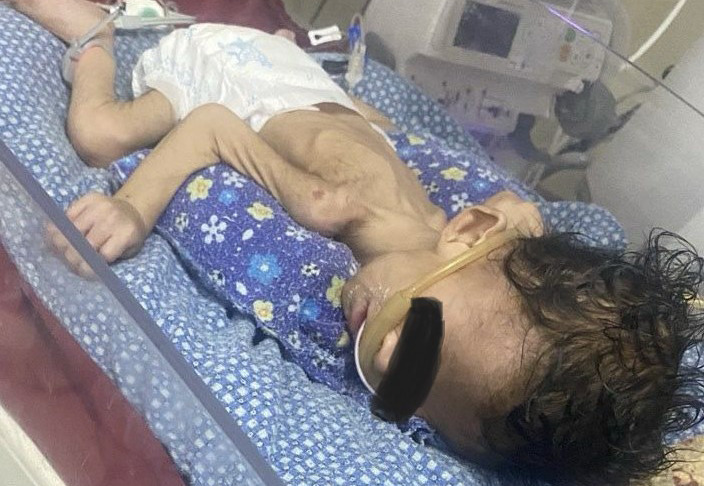
Ill-looking and cachexic child with Zellweger syndrome.

At 5 months of age, the patient's mother noticed noisy, fast breathing associated with chest recession, increased sweating and very poor feeding. The patient was referred to our hospital for pediatric intensive care unit admission. On examination, the general condition was ill-looking and cachexic (Figure 1). The pulse rate was 150 bpm, body temperature 99.1 degrees F, respiratory rate 60 per minute, oxygen saturation of 98% in NP and random blood sugar was 45 mg/dl. Anterior fontanelle was wide open, low set ear, hypertelorism, downward slanting of eyes, simian crease, cortical thumb and undescended testis were present on head-to-toe examination. The patient was admitted and treated with intravenous antibiotics like meropenem, clarithromycin, teicoplanin, fluconazole and IV dextrose for hypoglycemia. Even with prompt treatment and careful monitoring, the patient's condition kept deteriorating and later he developed severe sepsis with multi-organ failure leading to the death of the patient.

## DISCUSSION

Zellweger syndrome is a fatal autosomal recessive disorder, affected individuals have unusual physical features, severe neurological dysfunction, hepatorenal diseases and metabolic abnormalities.^5^ ZSDs are caused by variants in at least 13 PEX genes encoding PEX proteins involved in the biogenesis of peroxisomal membrane proteins and complete degradation of any peroxisomal remnants.^6^

Our patient presented with hypotonia, seizure, noisy breathing, and delayed swallowing reflex. Classically severe ZSD presents with hypotonia, epileptic seizure and failure to thrive which are thought to result from the accumulation of VLCFA and phytanic acid mainly in the nervous system; however, the specific pathogenic mechanism of these symptoms is poorly understood.^6^ Our patient also experienced severe hypotonia, developmental delay, muscle weakness, and feeding difficulty since birth due to which the patient had recurrent aspiration pneumonia complaining of noisy breathing and chest recession and was admitted to the local hospital several times.

Biochemically in ZSD, there is an increased level of serum very long chain fatty acid (VLCFA), phytanic acid, pristanic acid, liver transaminase and total bile acids.^6^ Our patient had an increased level of transaminase but the total bilirubin level was normal which is heterogeneity in biochemical findings. Using DNA from the patient's peripheral blood a whole genome sequencing was conducted at a lab in India where a homogenous missense variant in the PEX gene was detected.

Radiographically, an MRI of the brain at 2 months of age showed diffuse T2 high intensity in the subcortical and deep white matter of the bilateral cerebral hemisphere, probably an area of incomplete myelination which is a consistent finding of ZSD. MRI findings of the brain include developmental brain malformations like pachygyria, and polymicrogyria especially in the perisylvian region, periventricular neuronal heterotopia and poor myelination.^7^ In ZSD usually USG finding shows renal cortical cysts.^8^ But our patient had hydronephrotic changes which is heterogeneity in USG finding. There were multiple fenestrated ASDs and VSDs with left to right shunt.

The diagnosis is usually confirmed by finding raised phytanic acid and whole genome sequencing for the PEX gene along with classical clinical presentation in the patient. Currently, there is no cure for Zellweger syndrome or a standard course of treatment.^9^ In our case the patient presented with complications of pneumonia for which he was treated with intravenous antibiotics and fluids. Other causes of death in such patients include gastrointestinal bleeding and liver failure. Treatment is usually symptomatic and patients don't usually survive beyond 1 year of life.^9^

In conclusion, Zellweger syndrome is one of many dysmorphic syndromes which is a result of an inborn error of metabolism. Hence, a combined approach between the paediatrician, pediatric neurologist, clinical biochemist and geneticist is a key factor for the early recognition of this syndrome. This syndrome should be included in the differential diagnosis of infantile hypotonia with dysmorphism^9^.

## References

[1] Kaplan M, Eidelman AI, Shapira Y, Collins J, Goldfischer S (1988). Cerebrohepatorenal syndrome of Zellweger: a peroxisomal deficiency disorder. Case report and review.. Isr J Med Sci..

[2] Su L, Peng MZ, Chen XD, Wu S, Liu L (2024). Severe Zellweger spectrum disorder due to a novel missense variant in the PEX13 gene: a case report and the literature review.. Mol Genet Genomic Med..

[3] Lee PR, Raymond GV (2013). Child neurology: Zellweger syndrome.. Neurology..

[4] https://www.orpha.net/consor/cgi-bin/OC_Exp.php?lng=en&Expert=912.

[5] Walia A, Birath AL, Buchman CA (2023). Cochlear implantation and audiological findings in a child with Zellweger spectrum disorder.. Otolaryngology Case Reports..

[6] Gala F, Mathur A (2021). Neuro-imaging in Zellweger syndrome.. European society of Radiology..

[7] Bose M, Yergeau C, D'Souza Y, Cuthbertson DD, Lopez MJ, Smolen AK (2022). Characterization of severity in Zellweger spectrum disorder by clinical findings: a scoping review, meta-analysis and medical chart review.. Cells..

[8] Kheir AE (2011). Zellweger syndrome: a cause of neonatal hypotonia and seizures.. Sudan J Paediatr..

